# *Clostridium Butyricum* ZJU-F1 Benefits the Intestinal Barrier Function and Immune Response Associated with Its Modulation of Gut Microbiota in Weaned Piglets

**DOI:** 10.3390/cells10030527

**Published:** 2021-03-02

**Authors:** Jie Fu, Tenghao Wang, Xiao Xiao, Yuanzhi Cheng, Fengqin Wang, Mingliang Jin, Yizhen Wang, Xin Zong

**Affiliations:** 1 Key Laboratory of Molecular Animal Nutrition, Ministry of Education, College of Animal Sciences, Zhejiang University, Hangzhou 310058, China; fujie2019@zju.edu.cn (J.F.); 0619731@zju.edu.cn (T.W.); 21617021@zju.edu.cn (X.X.); 18905705528@zju.edu.cn (Y.C.); wangfq@zju.edu.cn (F.W.); mljin@zju.edu.cn (M.J.); 2 Key Laboratory of Animal Nutrition and Feed Science in Eastern China, Ministry of Agriculture, Hangzhou 310058, China

**Keywords:** *C. butyricum* ZJU-F1, microbiota, weaning piglets, intestinal barrier, TLR2

## Abstract

This study investigated the effects of dietary *C. butyricum* ZJU-F1 on the apparent digestibility of nutrients, intestinal barrier function, immune response, and microflora of weaned piglets, with the aim of providing a theoretical basis for the application of *Clostridium butyricum* as an alternative to antibiotics in weaned piglets. A total of 120 weanling piglets were randomly divided into four treatment groups, in which piglets were fed a basal diet supplemented with antibiotics (CON), *Bacillus licheniformis* (BL), *Clostridium butyricum* ZJU-F1 (CB), or *Clostridium butyricum* and *Bacillus licheniformis* (CB-BL), respectively. The results showed that CB and CB-BL treatment increased the intestinal digestibility of nutrients, decreased intestinal permeability, and increased intestinal tight junction protein and mucin expression, thus maintaining the integrity of the intestinal epithelial barrier. CB and CB-BL, as exogenous probiotics, were also found to stimulate the immune response of weaned piglets and improve the expression of antimicrobial peptides in the ileum. In addition, dietary CB and CB-BL increased the proportion of *Lactobacillus.* The levels of butyric acid, propionic acid, acetic acid, and total acid were significantly increased in the ceca of piglets fed CB and CB-BL. Furthermore, we validated the effects of *C. butyricum* ZJU-F1 on the intestinal barrier function and immune response in vitro and found *C. butyricum* ZJU-F1 improved intestinal function and enhanced the TLR-2-MyD88-NF-κB signaling.

## 1. Introduction

The intestinal tract, a very important organ of mammals, is not only the largest “gas station” and “sewage factory” in mammals, but also the largest “fortress” for disease prevention. Therefore, intestinal dysfunction will lead to a series of problems, such as diarrhea, which seriously threatens the health of humans, especially children, and even animals. Weaning is a common problem, which can lead to intestinal dysfunction in severe cases and can, in turn, induce severe diarrhea. Although antibiotics are usually prescribed for this problem, long-term use and abuse of subtherapeutic antibiotic doses can lead to dysfunction of the gut microbiota and the appearance of antimicrobial resistance, both of which have raised serious public health concerns [1,2]. For example, it was estimated that up to 14,000 Americans die each year from infection with *Clostridium difficile*, a drug-resistant bacterial strain that causes life-threatening diarrhea [3]. As a result, the European Union, USA, and China, which together raise more than 90 percent of the world’s pigs, banned antibiotics as feed additives in 2006, 2014, and 2020, respectively. Therefore, it is urgent to develop an antibiotic substitute that can replace antibiotics.

Probiotics, first identified in 1905, are viable nonpathogenic microorganisms that have been used to manipulate microorganisms in the host to improve measurable health outcomes [4]. Probiotics have been suggested as effective and promising agents for the treatment of inflammatory gastrointestinal barrier disorders, including infectious colitis, antibiotic-associated diarrhea, and inflammatory bowel diseases (IBD). Compared with antibiotics, probiotics will not cause intestinal microbiota disorder, but can also promote the normalization of intestinal flora by promoting various metabolic pathways of the body, so as to maintain the normal intestinal function. These organisms include *Escherichia coli* Nissle 1917 [5,6,7], *Lactobacillus fermentum* BR1 [8,9], and *Lactobacillus rhamnosus* GG (LGG) [10], all of which have been proven to play important roles in the treatment of intestinal inflammatory diseases. EcN1917 and LGG can significantly reduce diarrhea and increase diarrhea tolerance in infants and young children [11,12]. Existing research suggests that probiotics compete with pathogenic bacteria for mucosal adhesion sites, influence epithelial barrier functions and gut permeability, restore the commensal bacterial microbiota, increase secretion of short-chain fatty acids (SCFAs) and bacteriocins, and reduce the production of inflammatory cytokines in the intestine [13,14,15]. However, the exact functional mechanisms and modes of action are poorly understood.

*C. butyricum*, named for its capacity of producing high amounts of butyric acid, is a Gram-positive obligate anaerobic and spore-forming bacillus that has been used as a probiotic to treat and prevent diarrhea and intestinal microflora disorder in human beings and to enhance the humoral immune response [16,17,18]. In addition, *C. butyricum* has also been used well in chickens and rabbits [19,20]. We previously isolated a *C. butyricum* strain, named *C. butyricum* ZJU-F1, from the feces of a healthy swine and found that it could secrete high levels of bacteriocins and promote the growth of piglets [21]. However, the functions of *C. butyricum* ZJU-F1 on intestinal barrier function, immune response, and bacterial balance remain largely unexplored.

In this study, we aimed to investigate the reasons for dietary *C. butyricum* ZJU-F1′s ability to decrease the rate of diarrhea and increase nutrient digestibility. To do this, we used an antibiotic diet as the control and evaluated the effects of *C. butyricum* ZJU-F1 in weaned piglets on intestinal homeostasis, including barrier function, immune status, and the microbiota. Moreover, we established an in vitro model with porcine intestinal epithelial cells to investigate the mechanisms involved. This study revealed the function of *C. butyricum* ZJU-F1 on intestinal health and provided an alternative strategy to reduce the use of antibiotics.

## 2. Materials and Methods

### 2.1. Reagents

The *Clostridium butyricum* ZJU-F1 strain was initially isolated from the feces of a healthy nongenetically modified pig and was conserved in the China General Microbiological Culture Collection Center (CGMCC No.8939, Shanghai, China). *C. butyricum* ZJU-F1 was grown anaerobically in reinforced clostridium medium (RCM) at 37 °C with shaking at 200 rpm for 48 h. After centrifugation at 3000× *g* for 10 min at 37 °C, the cells were harvested and dried with drying technology at 60 °C for 48 h. The number of viable bacterial spores was determined by serially diluting the suspension on RCM agar(Haibo Biology, Qindao, China)and incubating at 37 °C for 24 h. The final concentration of *C. butyricum* in the animal diets was adjusted to 1.0 × 10^8^ viable spores/kg of diet. The *Bacillus licheniformis* (*B.licheniformis*) was a commercial product manufactured by Zhejiang Huijia Biological Technology Ltd. (Huzhou, China). This product is composed of spray-dried spore-forming *B. licheniformis* endospores and contains at least 1.0 × 10^9^ viable spores/kg diet.

### 2.2. Animals and Experiment Design

The animal experimental protocols were approved by the Animal Care and Use Committee of Zhejiang University. A total of 120 weaned piglets of similar age (Duroc × Landrace × Yorkshire) were randomly allotted to four treatments based on initial body weight. There were three replicate pens in each treatment with 10 pigs per pen (5 sows and 5 boars) and the dietary treatments are shown in Table 1. The experiment lasted for 14 days with 6 piglets slaughtered in each group at the end of experiment. The pigs were fed a corn–soybean meal-based diet that contained no antibiotics or growth-promoting concentration of zinc. All basal diets met or exceeded the nutrient requirements as suggested by the National Research Council (NRC) and their compositions are shown in Table 2.

### 2.3. Nutrient Digestibility, Digestive Enzyme Activity, and Intestinal Morphology Assessment

Nutrient digestibility was calculated as described by Wang et al. [22]. For the digestive enzyme activity of duodenum and jejunum determination, the mucosa of the duodenum and jejunum were collected and homogenized by adding sterile 4% saline solution to prepare 10% (W:V) homogenates. The homogenate was centrifuged at 1000× *g* for 10 min at 4 °C, then each digestive enzyme activity (protease, amylase, and lipase) in the supernatant was determined by spectrophotometry using a commercial kit according to the manufacturer’s instruction (Sigma-Aldrich Co, Saint Louis, MO, USA). The middle duodenum was collected and fixed in 10% PBS-buffered formalin and embedded in paraffin. The 0.2 μm thick paraffin sections were cut and stained with hematoxylin and eosin (H&E). Images of the H&E-stained sections were acquired through a Leica DM3000 Microsystem (Leica, Germany). Then, the morphology of the duodenum was evaluated by scanning electron microscopy (SEM, TM-1000, Hitachi, Japan) and transmission electron microscopy (TEM, H-7650, Hitachi, Japan) as previously described [23]. SEM and TEM images were acquired and analyzed using a Hamamatsu ORCA-HR digital camera system operated with AMT software (Advanced Microscopy Techniques Corp., Danvers, MA, USA) and Semicaps 2000 software, respectively.

### 2.4. Western Blot Analysis and Antibodies

Total protein was extracted from separate samples of piglet jejunum and ileum and intestinal epithelial cells (IPEC-J2) using a Total Protein Extraction Kit (KeyGen Biotech, Nanjing, China). Protein concentrations were quantified using the BCA assay kit (KeyGEN Biotech, Nanjing, China). Equivalent amounts of protein were first separated by SDS-PAGE, electroblotted onto PVDF membranes, and blocked with 5% nonfat milk for 1 h at room temperature. After blocking, the PVDF membranes were incubated overnight at 4 °C with primary antibodies, followed by washing with Tris-buffered saline containing Tween (TBST) three times for 10 min each. Subsequently, the membranes were incubated with secondary antibodies for 1 h at room temperature. The membranes were then washed with TBST as before. Specific bands were visualized with an ECL detection kit (Santa Cruz Biotechnology, Inc., CA, USA). Antibodies to the following were used in this study: ZO-1 (21773-1-AP, Rabbit), Occludin (27260-1-AP, Rabbit), Claudin-1 (13050-1-AP, Rabbit), TLR-2 (Proteintech, Rosemont, IL, USA, 66645-1-Ig), MyD88 (Proteintech, 23230-1-AP), NF-κB (Proteintech, 10745-1-AP), GAPDH (60004-1-Ig, Rabbit).

### 2.5. RNA Isolation and qRT-PCR

Isolation of total RNA and cDNA synthesis by reverse transcription was performed using the TRIzol reagent and a reverse transcriptase kit (Thermo Fisher Scientific, Boston, MA, USA). The cDNA was quantified using a Nanodrop ND-1000 Spectrophotometer (Nanodrop Technologies, Inc. Wilmington, DE, USA) and mRNA expression was quantitated using the SYBR Green quantitative PCR system. Primers for amplifying each target gene are listed in Table 3 and all data were analyzed using the 2^−ΔΔCt^ method.

### 2.6. Plasma D-Lactate and DAO Levels

After slaughter, 10 mL of venous blood was collected. The concentrations of D-lactic acid and the activity of diamine oxidase (DAO) were measured by enzymatic spectrophotometry using a commercial kit (Jiancheng Bioengineering Institute of Nanjing, China), according to the manufacturer’s instructions.

### 2.7. Cell Culture

The porcine intestinal epithelial cell line IPEC-J2 was obtained from the Cell Bank of the Chinese Academy of Sciences (Shanghai, China) and cultured in DMEM-F12 medium (Youcon, Beijin, China) supplemented with 10% (*v*/*v*) fetal bovine serum (Gibco, California, USA) and antibiotics (100 U/mL penicillin and 100 μg/mL streptomycin sulfate) at 37 °C in a humidified incubator under 5% CO2 [21,24].

### 2.8. Cell Damage and Adherence Assay

IPEC-J2 cells were removed with trypsin and were inoculated into a 48-well plate (Coring, NY, USA) for culture. When the cells reached 60%~70% confluence, they were stimulated with the pathogenic enterotoxigenic Escherichia coli (ETEC) K88 and *C. butyricum* ZJU-F1 for 1.5 h, separately, with four replicates per treatment. At the end of the stimulation, the culture medium was collected and lactate dehydrogenase (LDH) activity was detected with an LDH kit (Roche Applied Science). Assays for adherence, competition, exclusion, and displacement were performed as previously described [25].

### 2.9. Diversity Analysis of Cecal Microorganisms

Total bacterial DNA was extracted with a fecal genomic DNA extraction kit (Solarbio, Beijing, China). The extraction quality was determined by 1% agar-gel electrophoresis according to the instructions. The pure DNA was stored at −80 °C. Then, 16S rDNA was amplified by PCR with universal primers in the V3-V4 region (338F: ACTCCTACGGGAGGCAGCA, 806R: GGACTACHVGGGTWTCTAAT). PCR was conducted as follows: pre-denaturation at 95 °C for 3 min, denaturation at 95°C or 25 s, annealing at 55 °C for 25 s, stretching at 72 °C for 45 s, 25 cycles, and a final stretching at 72 °C for 10 min. The AxyPrep DNA gel extraction kit (Axygen Biosciences, Union City, CA, USA) and QuantiFluor-ST instrument (Promega, USA) were used to further extract, purify, and quantify the PCR products. The MiSeq platform (Shanghai Majorbio Biopharm Technology Co., Ltd.) was used to describe the bacterial community based on the gene segment from the V3-V4 portion of the 16S rRNA gene. PE reads obtained by MiSeq sequencing were splintered according to overlap relation to form a complete DNA sequence. The effective sequence was obtained by distinguishing the samples according to the primer sequence label and primer label, and the sequence direction was corrected. Meanwhile, Trimmomatic and Flash software were used for sequence quality control and filtration. OTUs were clustered with 97% similarity cutoff using UPARSE (version 7.1). Mothur 1.30.1 software was used to analyze the microbial diversity in the samples with outcome indexes Chao1, Shannon, and Coverage. Qiime1 software was used to calculate the community similarity of samples and principal component analysis (PCA) was conducted through the ecological distance between samples. The RDP classifier algorithm (http://rdp.cme.msu.edu/) against the Greengenes 16S rRNA database was used with a confidence threshold of 70%.

### 2.10. Determination of Short-Chain Fatty Acids (SCFAs)

SCFAs were determined as previously described [26]. Briefly, the cecal contents were suspended in PBS, then ultrasonicated and centrifuged at 12,000× *g* for 10 min at 4 °C. Then 25% metaphosphate and 210 mmol/L cortical acid were added to the supernatant, followed by 30 s of vortex and 10 min of centrifugation (5000 r/min at 4 °C). The supernatant was taken out and methanol was added (volume ratio, supernatant:methanol = 1:3), then vortexed for 30 s and centrifuged at 1000 r/min for 10 min. The supernatant was then filtered through filter paper for HPLC detection. A PrevailTM Organic Acid column (250 mm × 4.6 mm) was used with the following detection conditions: temperature, 40 °C; wavelength, 217 nm; pressure, 0.1–4000 psi.

### 2.11. Statistical Analysis

All statistical analyses were performed using SPSS software (Version 20.0, IBM Corp, Armonk, NY, USA) and all data were expressed as means ± standard error (SEM). Significant differences between the control and experimental groups were determined by a one-way ANOVA with a Duncan multiple. Statistically, a *p*-value of less than 0.05 was considered significant.

## 3. Results

### 3.1. Effects of C. Butyricum ZJU-F1 on Diarrhea Rate, Apparent Digestibility, and Digestive Enzymes Activity

**Figure 1:**
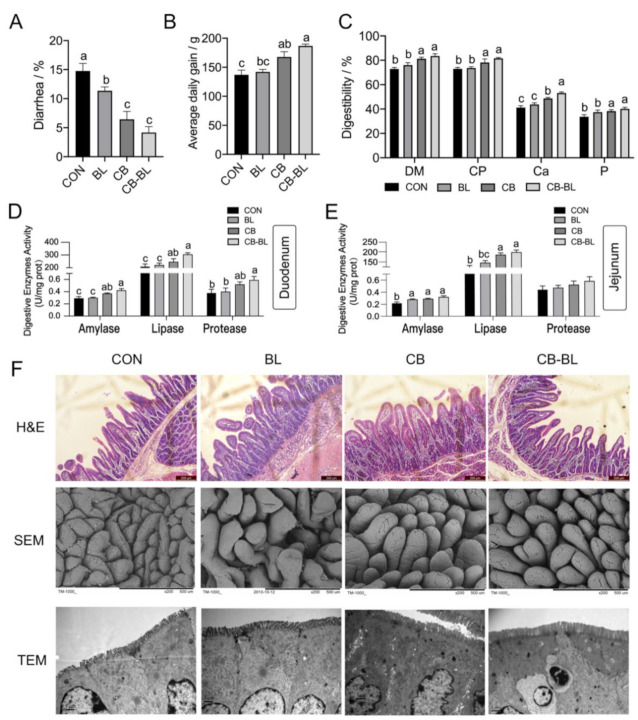
Effects of *C. butyricum* ZJU-F1 on diarrhea rate, nutrients digestibility, digestive enzymes activity, and intestinal morphology. (**A**–**C**) The diarrhea rate (**A**), average daily gain (**B**), nutrients digestibility (**C**), and digestive enzymes activity of duodenum and jejunum (**D**–**E**) of piglets fed each diet. The data are expressed as the mean ± SEM; bars with different small capital letters are statistically different from one another. *n* = 30 (A–B), *n* = 6 (C–E) biological replicates. (**F**) Intestinal morphology of duodenum sections. Representative H&E-stained section from duodenum (top; bars, 200 μm), SEM images (middle; bars, 500 μm), TEM images (bottom; bars, 1 μm).

Our previous study [21] showed that dietary *C. butyricum* ZJU-F1 (CB) significantly increased the growth performance and decreased the diarrhea rate. These effects were more marked when in combination with *B. licheniformis* (CB-BL) (Figure 1A–B). However, no significant difference was found between the BL group and the CON group. We found further differences in the piglets in the BL group, specifically, digestion of dry matter (DM), crude protein (CP), Ca, and P of piglets in the CB or CB-BL groups were significantly greater (Figure 1C).

The improvement of apparent digestibility is usually closely related to the activity of digestive enzymes in the intestine, so we examined the activity of amylase, lipase, and protease in the duodenum and jejunum. Similar to the results of apparent digestibility, the digestive enzyme activity of the CB group or CB-BL group was significantly higher than that of group BL and CON (Figure 1D–E). These results indicated that dietary *C. butyricum* ZJU-F1 was beneficial to the intestinal digestive function of weaned piglets, especially when used in combination with *B. licheniformis*.

### 3.2. Effects of C. Butyricum ZJU-F1 on Intestinal Morphology

To explore the reasons for the improvement of digestive function in weaned piglets by *C. butyricum* ZJU-F1, we evaluated the effects of *C. butyricum* ZJU-F1 on the intestinal barrier function. The data showed that villous height in the jejunum of the CB and CB-BL groups increased significantly compared with the CON group (Figure 1F, top); this result was confirmed by SEM at 150× magnification (Figure 1F, middle). In addition, surface damage to villi in the jejunum was alleviated by *B. licheniformis* treatment (Figure 1F, middle). Furthermore, the height of microvilli in the jejunum of piglets fed with *C. butyricum* ZJU-F1, *B. licheniformis*, and a combination of the two, was much higher than that in the piglets fed CON diets (Figure 1F, bottom). These results suggest that *C. butyricum* ZJU-F1 improved intestinal morphology and integrity in weaned piglets with clinical diarrhea.

### 3.3. Effects of C. Butyricum ZJU-F1 on Intestinal Barrier Function

**Figure 2:**
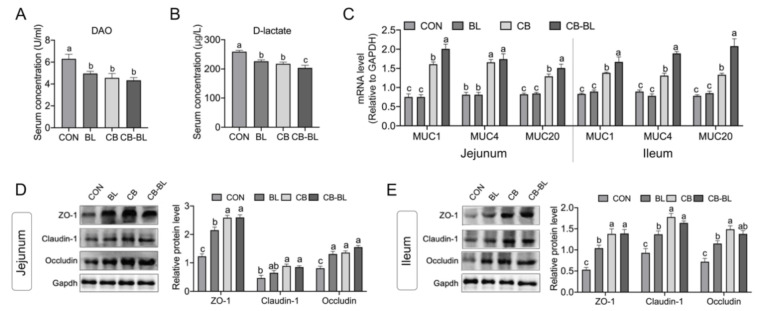
Effects of *C. butyricum* ZJU-F1 on intestinal barrier function. (**A**–**B**) The concentration of diamine oxidase (**A**) and D-lactic acid (**B**) in serum of piglets fed each diets. (**C**) qRT-PCR quantified *muc1*, *muc4*, and *muc20* mRNA abundance in jejunum (left) and ileum (right). The results were presented relative to those of Gapdh. (**D**–**E**) Western blot analysis of the expression of tight junction proteins in jejunum (**D**) and ileum (**E**). The right panel shows the relative levels quantified by densitometry and normalized to Gapdh. The data are expressed as the mean ± SEM, *n* = 6, biological replicates; bars with different small capital letters are statistically different from one another.

Plasma D-lactate and DAO levels are endogenous markers that indicate changes in permeability, which could directly reflect the degree of damage to the intestinal epithelial mucosa [27]. As a direct validation, we determined the levels of DAO and D-lactate in each group. As shown in Figure 2A,B, no matter whether the piglets were fed with *C. butyricum* ZJU-F1, *B. licheniformis*, or both, the serum level of DAO and D-lactate decreased significantly, which indicated that the intestinal integrity was enhanced. Compared with BL, CB-BL significantly reduced D-lactate, which indicated that this was the effect of *C. butyricum* ZJU-F1. These results suggest that supplementation of diets with *C. butyricum* ZJU-F1 can significantly decrease intestinal permeability.

To further illustrate the effects of *C. butyricum* ZJU-F1 on intestinal barrier function, we analyzed the expression of mucins and tight junction proteins. Mucin, secreted by intestinal epithelial cells and goblet cells, can mix with water and electrolytes on the intestinal mucosal surface to form a chemical barrier on the intestinal epithelium to protect the intestinal mucosa from chemical and mechanical damage [28,29]. Interestingly, we found that mRNA expression of mucins (MUC1, MUC4, and MUC20) in the CB and CB-BL groups was significantly increased, but *B. licheniformis* treatment alone had barely any effect on the mucins’ expression in the jejunum (Figure 2C, left). Similar observations were also made in the ileum (Figure 2C, right). In addition, we also evaluated the expression of the intestinal tight junction proteins ZO-1, claudin-1, and occludin. As expected, we found that the three tight junction proteins were significantly increased in both the jejunum and ileum of piglets treated with *C. butyricum* ZJU-F1, *B. licheniformis*, or both (Figure 2D,E). In addition, compared with BL, CB-BL significantly increased the expression of mucin and tight junction proteins, which indicated that this was the effect of *C. butyricum* ZJU-F1.

### 3.4. Effects of C. Butyricum ZJU-F1 on the Intestinal Immune Response

**Figure 3:**
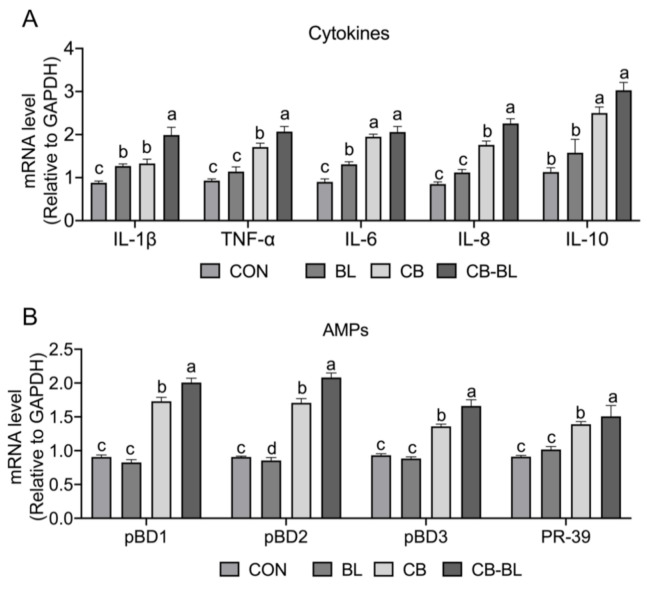
Effects of *C. butyricum* ZJU-F1 on intestinal immune response. (**A**) qRT-PCR quantified mRNA abundance of cytokines, including *IL-1β, TNF-α, IL-6, IL-8*, and *IL-10*, in jejunum of piglets fed each diet. (**B**) The mRNA level of antimicrobial peptides, including *pBD1, pBD2, pBD3,* and *PR-39* from the same sample of (**A**). The results were presented relative to those of Gapdh. The data are expressed as the mean ± SEM, *n* = 6, biological replicates; bars with different small capital letters are statistically different from one another.

As inflammation is one of the primary factors responsible for the destruction of the epithelial barrier [30], we analyzed the expression of proinflammatory cytokines. Interestingly, we observed that, after treatment with BL, CB, and CB-BL, the expression of the proinflammatory factors IL-1β, TNF-α, IL-6, and IL-8 was significantly increased, while the expression of the anti-inflammatory cytokine IL-10 was also upregulated compared to the control group (Figure 3A). Additionally, we also detected the expression of endogenous antibiotic peptides. Piglets treated by CB and CB-BL upregulated the gene expression of pBDs and PR-39 compared to the control group, but BL treatment did not improve their expression (Figure 3B). In addition, compared with BL, CB-BL significantly increased the expression of cytokines (IL-1β, TNF-α, IL-8, and IL-10) and antimicrobial peptides, which indicated that this was the effect of *C. butyricum* ZJU-F1.

### 3.5. Effects of C. Butyricum ZJU-F1 on Intestinal Microbial Diversity

**Figure 4:**
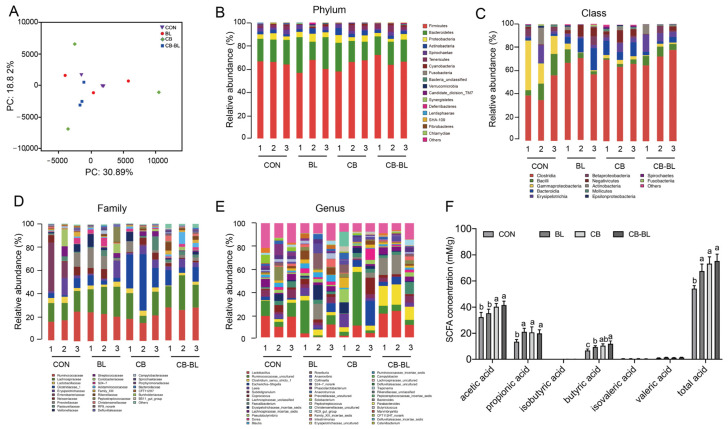
Changes in gut microbial community and SCFAs in weaning piglets after probiotics treatment. (**A**) Multiple sample PCA analysis of bacterial in weaned piglets fed each diets; purple inverted triangle, CON; red circle, BL; green diamond, CB; blue square, CB-BL. (**B**) Microbial community bar plot by phylum. (**C**) Microbial community bar plot by class. (**D**) Microbial community bar plot by family. (**E**) Microbial community bar plot by genus. (**F**) Changes of SCFAs concentrations in cecal contents of piglets fed. The data are expressed as the mean ± SEM, *n* = 3 (**A**–**E**), *n* = 6 (**F**), biological replicates; bars with different small capital letters are statistically different from one another.

Intestinal flora plays an important role in the digestion and absorption of nutrients in the body, and it is also closely related to the metabolism, physiological status, and health of the host. Here, we analyzed the intestinal microbial diversity in the ceca of each group. The PCA plot showed that the distribution between CON and CB-BL was relatively discrete, indicating that CB-BL treatment had a significant effect on the distribution of cecal microorganisms in weaned piglets. However, analysis of the phyla indicated that there were no significant differences in the dominant bacteria, including *Firmicutes*, *Bacteroidetes*, and *Proteobacteria* (Figure 4B). We found that the BL, CB, and CB-BL treatments increased the percentage of *Clostridia* but decreased the percentage of *Bacilli* and *Gammaproteobacterial* at the class level (Figure 4C). Furthermore, we observed that CB increased the percentage of *Clostridiaceae_1* at the family level (Figure 4D). As shown in Figure 4E and Table 4, we further found that the main bacteria in the cecal contents of weaned piglets were *Ruminococcaceae_uncultured*, *Clostridium_sensu_stricto_1*, *Lactobacillus, Leeia, Faecalibacterium, Coprococcus, Pseudobutyrivibrio* at the genus level. In contrast to the control group, the CB-BL diet increased the percentage of *Ruminococcaceae_uncultured*, *Lactobacillus, Streptococcus* in *Firmicutes* in which *Lactobacillus* was significantly increased. In addition, there were significant differences in cecal *Lactobacillus* between the BL and the CB-BL as an effect of CB (Table 4).

### 3.6. Effects of C. Butyricum ZJU-F1 on Cecal SCFAs

SCFAs play important roles in the intestinal flora [31]. We, therefore, determined the SCFAs present in each group. As shown in Figure 4F, compared to the CON group, the secretions of acetic, propionic acid, butyric acid, and total acid in the cecal contents of weaned piglets treated with BL, CB, and CB-BL were significantly increased. However, the concentrations of acetic acid, propionic acid, and butyric acid in the CB and CB-BL groups were higher than those in the BL group. In addition, there were significant differences in acetic acid and butyric acid between the BL and the CB-BL as an effect of CB. These results showed that *C. butyricum* ZJU-F1 diets can increase the concentrations of acetic acid, propionic acid, and butyric acid in the cecal contents of weaning piglets, which may be related to the regulation of intestinal microflora.

### 3.7. C. Butyricum ZJU-F1 Enhances the Intestinal Barrier Function and TLR-2-MyD88-NF-κB Signaling Pathway

**Figure 5:**
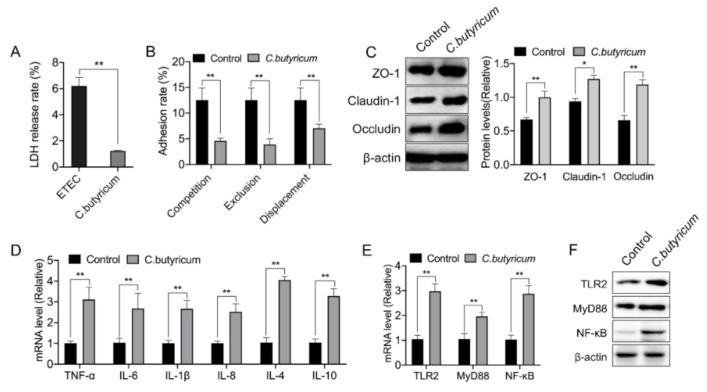
*C. butyricum* ZJU-F1 enhances the intestinal barrier function and TLR-2 signaling. (**A**) LDH release of IPEC-J2 treated with or without *C. butyricum* ZJU-F1. (**B**) Competition, exclusion, and displacement of ETEC K88 adhering to IPEC-J2 cells with *C. butyricum*. (**C**) Western blot analysis of the expression of tight junction proteins in the IPEC-J2 cells treated by *C. butyricum* ZJU-F1. The right panel shows the relative levels quantified by densitometry and normalized to β-actin. (**D**) qPCR analysis of the expression of cytokines, including TNF-α, IL-6, IL-1β, IL-8, IL-4, and IL-10, in IPEC-J2 treated with *C. butyricum* ZJU-F1. (**E**) qRT-PCR analysis of the expression of TLR2, MyD88, and NF-κB in the IPEC-J2 cells treated by *C. butyricum* ZJU-F1. (**F**) Western blot analysis of the expression of TLR2, MyD88, NF-κB from the same sample of (E). The data are expressed as the mean ± SEM, * *p* < 0.05, ** *p* < 0.01; *n* = 6, biological replicates.

To better understand the precise mechanism underlying the regulation of the intestinal barrier function by *C. butyricum* ZJU-F1, we established an in vitro model and selected the pathogenic enterotoxigenic Escherichia coli (ETEC) K88 as a representative of the many harmful bacteria in the gut. We first noted that the damage caused by *C. butyricum* ZJU-F1 to IPEC-J2 cells was significantly lower than that caused by ETEC K88, which was assessed by the LDH release rate (Figure 5A). The results of the competition test showed that *C. butyricum* ZJU-F1 could significantly inhibit the adhesion of ETEC K88 to IPEC-J2 cells, reducing the adhesion rate of ETEC K88 from 12.52% to 4.59%, when *C. butyricum* ZJU-F1 and ETEC K88 were incubated simultaneously with the cells (Figure 5B). Additionally, exclusion and displacement tests also indicated a significant reduction in the adhesion of ETEC K88 to IPEC-J2cells, in which the effect of exclusion was more significant (Figure 5B).

Then, we verified that *C. butyricum* ZJU-F1 could significantly promote the expression of the tight junction proteins ZO-1, claudin-1, and occludin in IPEC-J2 cells (Figure 5C). We found that *C. butyricum* ZJU-F1 significantly upregulated the gene expression of cytokines, including TNF-α, IL-1β, IL-6, IL-8, IL-4, and IL-10 (Figure 5D), which was consistent with the results in vivo. Moreover, our results showed that *C. butyricum* ZJU- F1 significantly upregulated the gene expression of TLR2, MyD88, and NF-κB (Figure 5E).

## 4. Discussion

In the study, we found that *C. butyricum* ZJU-F1 had beneficial effects on the physiological functions of the small intestine and significant effects on the expression of cytokines and AMPs and cecal *Lactobacillus*, acetic acid, and butyric acid. Moreover, we further confirmed in vitro that *C. butyricum* ZJU-F1 improves intestinal barrier function and found that it enhances the TLR-2-MyD88-NF-κB signaling pathway.

During weaning, the activity of intestinal digestive enzymes decreases, the mucosal morphology and structure change, and the permeability increases, resulting in intestinal barrier dysfunction together with decreased digestion and absorption, in turn leading to intestinal diseases and diarrhea, hindering piglet growth [32]. Probiotics can affect the activity of some endogenous digestive enzymes and nutrients digestibility in the host, but there are few reports about weaning piglets. Our results indicated that dietary *C. butyricum* ZJU-F1 was beneficial to the physiological functions of the small intestine, digestive enzymes activity, and digestive function of weaned piglets. This might be partly due to *C. butyricum* ZJU-F1 promoting the secretion of SCFAs in the small intestine and improving the gut microbiota, thus improving the activity of digestive enzymes, and finally resulting in the improvement of the digestion and absorption of nutrients.

Intestinal barrier function is formed by a single layer of intestinal epithelial cells bound by tight junction proteins, which separates the coelenterate from the internal environment and restricts the spread of pathogens, toxins, and allergens from the lumen to the circulatory system. Plasma D-lactate is a metabolic product fermented by bacteria in the gut and DAO is an enzyme in the intestinal mucosal upper villi cells. Under normal conditions, there are little or no D-lactate and DAO in the blood, which are released into the blood only when the intestinal mucosal barrier is damaged, so their levels could directly reflect the degree of damage to the intestinal epithelial mucosa and permeability [27]. In addition, the higher the permeability, the greater the loss of nutrients in the gut [33,34]. Tight junctions, closely related to nutrient absorption and resistance to disease-causing microorganisms, are the main type of connection in intestinal epithelial cells [35]. In recent years, the effect of probiotics on intestinal barrier function has attracted much attention. It has been reported that the growth performance improvement *of C. butyricum* was associated with reducing or preventing the barrier damage caused by ETEC K88 in piglets and broiler chickens [36,37]. Zhang et al. [37] found that oral administration of *C. butyricum* can significantly decrease intestinal permeability challenged with ETEC K88 in broiler chickens. *Lactobacillus reuteri* increased the expression of Claudin-1, Zo-1, and occludin in the intestinal epithelium of newborn piglets, and reversed the barrier function damage of IPEC-J2 cells in the intestinal epithelium of pigs caused by LPS. As expected, our results suggest that *C. butyricum* ZJU-F1 enhances the expression of intestinal tight junction proteins in vivo and in vitro, and improves intestinal barrier function in vivo, decreasing plasma D-lactate and DAO levels. Taken together, these findings show that the enhancement of nutrient digestion and absorption in weaned piglets treated with *C. butyricum* ZJU-F1 might be partly due to the reduction of intestinal permeability and the improvement of intestinal barrier function.

A large number of microbes live in the intestinal tracts of animals. The host provides sufficient nutrition and a stable environment for the intestinal microbial microbiota. In turn, microbes participate in the digestion, absorption, and energy supply of the host, while also regulating the host’s physiological functions [38]. Many studies have shown that probiotics can regulate the balance of intestinal microbiota in piglets, contributing to the formation of intestinal microflora dominated by beneficial bacteria, and thus mediating physiological functions such as nutrient metabolism, immunity, and disease in animals. Zhang et al. [39] showed that dietary supplementation of *C. butyricum* dramatically increased *Selenomonadales* and decreased *Clostridiales*, and within *Selenomonadales, Megasphaera* became the dominant genus, increasing from 3.79 to 11.31%. Our results suggested that CB and CB-BL could slightly increase the percentage of *Firmicutes,* but with no significance, in the cecum of weaning piglets and slightly reduce the number of *Bacteroidetes*, but with no significance. Upon further analysis of the genus level, the results showed that CB and CB-BL can increase the proportion of *Lactobacillus* in the cecum contents. However, changes in gut microbes can significantly alter SCFAs in the intestine.

SCFAs, the primary end products of bacterial fermentation and the main energy source of intestinal epithelial cells, can bind with varying affinities to G protein receptors in the intestines and other cells to regulate energy metabolism, intestinal homeostasis, and immune responses [38,40,41]. Wang et al. found that supplementation of *C. butyricum* can increase the concentrations of acetic acid, propionic acid, and butyric acid in the colon of weaning piglets [42]. We found that there were significant differences in acetic acid and butyric acid between the BL and the CB-BL as an effect of CB. The difference in cecal *Lactobacillus* between the BL and the CB-BL was also significant. By balancing the intestinal microbiota, *C. butyricum* can accelerate the digestion and utilization of nutrients by intestinal microorganisms and thus ferment more SCFAs, thus positively regulating the metabolism of the body and thus covering the function of the small intestine. Therefore, we have a hypothesis that the improvement of physiological function in the small intestine of *C. butyricum* ZJU-F1 might be partly due to alterations in microbial-derived metabolites. However, the level of SCFAs is lower in the control group, and one possibility is that antibiotics reduce SCFAs by destroying intestinal microbiota. If antibiotics are not fed, the piglets will be infected with pathogenic bacteria, which will also cause the colonization of harmful bacteria in the intestinal tract, destroy the intestinal microbiota, and thus reduce SCFAs. Therefore, we believe that in order to evaluate this possibility, healthy piglets should be used to feed antibiotics or *C. butyricum* ZJU-F1 to in the future.

Among the diarrhea of newborn piglets caused by pathogenic bacteria, the diarrhea caused by ETEC K88 is the most serious. After ETEC invades the body, it secretes adhesin that helps it adhere to and colonize intestinal epithelial cells. Intestinal peristalsis or intestinal secretions make it difficult to remove ETEC K88, thus the ETEC K88 will absorb nutrients in the intestinal tract and rapidly reproduce, producing enterotoxin, causing diarrhea of piglets, and even leading to death of piglets [43]. Therefore, to better understand the precise mechanism underlying the regulation of the intestinal barrier function by *C. butyricum* ZJU-F1, we established an in vitro model and selected the ETEC K88 as a representative of the many harmful bacteria in the gut. Our results confirmed that *C. butyricum* ZJU-F1 could significantly reduce the adhesion of ETEC K88 to IPEC-J2. When *C. butyricum* ZJU-F1 adhered preferentially, the adhesion rate of ETEC K88 decreased to 3.89%. When the ETEC K88 adhered preferentially, *C. butyricum* ZJU-F1 could replace the adhesion of ETEC K88 with a decrease of adhesion rate from 12.52% to 7.44%. This may be because *C. butyricum* ZJU-F1 occupies the adhesion site of ETEC K88 or stimulates the secretion of mucin by IPEC-J2 cells to change the composition and characteristics of mucus, thereby reducing the adhesion and colonization of ETEC K88 to intestinal epithelial cells. In addition, acetic acid and butyrate secreted by *C. butyricum* ZJU-F1 can also kill or inhibit the growth and reproduction of pathogenic microorganisms.

*C. butyricum* not only has direct nutritional function, but also can stimulate intestinal mucosal immune response in animals [39]. Our results showed that the expression of proinflammatory cytokines TNF-α, IL-1β, IL-6, and IL-8, and anti-inflammatory cytokines IL-4 and IL-10 were significantly upregulated after *C. butyricum* ZJU-F1 treatment in IPEC-J2, consistent with the results of animal experiments. In general, animal or cell infection with pathogenic bacteria is detrimental, but the release of proinflammatory cytokines in the body after infection is a way to resist pathogenic bacteria. An appropriate amount of proinflammatory cytokines can regulate the immune response to some extent, resist or clean pathogen infection, and have the effects of promoting the repair of damaged tissues and causing tumor cell apoptosis, and so forth, but excessive amounts of proinflammatory cytokines can cause intestinal tissue damage and destroy the immune balance of the body [44]. In weaning piglets and weaning rex rabbits, studies have shown that *C. butyricum* weakly activates the body’s immune response [19,45], and alleviates high-fat diet-induced steatohepatitis in mice via enterohepatic immunoregulation [46]. We believe that the CB group has a low level of immune response, which may be a self-protective mechanism of the immune system that has evolved, helping the body to clear the pathogen, thus contributing to the healthy growth of animals. Mechanistically, we found that *C. butyricum* ZJU-F1 can significantly increase the expression levels of TLR2, MyD88, and NF-κB, indicating that *C. butyricum* ZJU-F1 may activate the TLR-2-dependent Myd88/NF-κB signaling pathway. Consistently, the notion that the presence of TLR2 is required for *C. butyricum* to activate the immune response has been further confirmed by Gao et al. in HT-29 cells [18] and Sui et al. in Cajal cells [47]. Therefore, we speculated that *C. butyricum* ZJU-F1 may regulate the expression of proinflammatory and anti-inflammatory factors in intestinal epithelial cells by activating the TLR-2-dependent NF-KB signaling pathway of MyD88, thus playing a role in the immune regulation of intestinal mucosa.

## 5. Conclusions

In this study, we aimed to explore the mechanism underlying dietary *C. butyricum* ZJU-F1′s ability to decrease the rate of diarrhea and increase nutrient digestibility. The results showed that *C. butyricum* ZJU-F1 treatment increased the intestinal digestibility of nutrients, decreased intestinal permeability, and increased intestinal tight junction protein and mucin expression, thus maintaining the integrity of the intestinal epithelial barrier. These effects were more marked when in combination with *B. licheniformis*. In addition, dietary *C. butyricum* ZJU-F1 increased the proportion of *Lactobacillus*, which was accompanied by the increase of butyric acid and propionic acid. Mechanistically, we found *C. butyricum* ZJU-F1 promoted the improvement of intestinal function, and the enhancements of the TLR-2-MyD88-NF-κB signaling pathway and SCFA production in cecal may be involved.

## Figures and Tables

**Table 1:** Dietary supplementation of 4 different treatments based on the basal diet.

Item	Control	BL	CB	CB-BL
Zinc (mg Zn (ZnO)/kg)	1.125	1.125	1.125	1.125
Antibiotics ^a^ (mg/kg)	170	-	-	-
*C. butyricum* ZJU-F1 (CFU/kg)	-	-	10^8^	10^8^
*Bacillus licheniformis* (CFU/kg)	-	10^9^	-	10^9^

^a^ The antibiotics were composed of 100 mg olaquindox/kg, 20 mg colistin sulfate/kg, and 50 mg kitasamycin/kg.

**Table 2:** Ingredient composition and nutrient levels of the basal diet.

Item (Ingredient (g/kg))	Content	Item (Nutrient Levels) ^b^	Content
Maize	567	Digestible energy (MJ/kg)	13.98
Extruded soybean	130	Crude protein (g/kg)	191
Soybean meal	155	Moisture (g/kg)	110.3
Sucrose	10	Lys (g/kg)	13.5
Fish meal	30	Met (g/kg)	3.0
Dried whey	30	Ca (g/kg)	9.3
Spay-dried plasma protein	10	P (g/kg)	6.5
Monocalcium phosphate	10		
Limestone	8		
Soybean oil	10		
Vitamin-trace mineral premix ^a^	40		

^a^ Provided per kilogram of diet: 16,000 IU vitamin A, 4000 IU vitamin D3, 100 IU vitamin E, 0.5 mg vitamin K3, 2 mg vitamin B1, 4.5 mg vitamin B2, 7 mg vitamin B6, 0.03 mg vitamin B12, 0.2 mg biotin, 10 mg folic acid, 30 mg nicotinic acid, 22 mg pantothenic acid; 85 mg Fe (FeSO4), 100 mg Cu (CuSO4), 0.3 mg Mn (MnSO4), 0.14 mg I (CaI2). ^b^ The data regarding crude protein, moisture, Ca, and P are determined values, the others are calculated values.

**Table 3:** Specific primers used for real-time PCR.

Gene	Forward Primer (5′→3′)	Reverse Primer (5′→3′)
MUC1	ACACCCATGGGCGCTATGT	GCCTGCAGAAACCTGCTXAT
MUC4	GATGCCCTGGCCACAGAA	TGATTCAAGGTAGCATTCATTTGC
MUC20	AGGCAGTTACAACATCCACAGAAG	CTGTAGACCATGGCCGAGAAC
TNF-α	CCAATGGCAGAGTGGGTATG	TGAAGAGGACCTGGGAGTAG
IL-6	TGGCTACTGCCTTCCCTACC	CAGAGATTTTGCCGAGGATG
IL-1β	ACAAAAGCCCGTCTTCCTG	ATGTGGACCTCTGGGTATGG
IL-4	GGACACAAGTGCGACATCA	GCACGTGTGGTGTCTGTA
IL-8	TTCGATGCCAGTGCATAAATA	CTGTACAACCTTCTGCACCCA
IL-10	CAGATGGGCGACTTGTTG	ACAGGGCAGAAATTGATGAC
PR-39	CAAGGCCACCTCCGTTTT	CCACTCCATCACCGTTTTCC
pBD1	TTCCTCCTCATGGTCCTGTT	AGGTGCCGATCTGTTTCATC
pBD2	TGTCTGCCTCCTCTCTTCC	AACAGGTCCCTTCAATCCTG
pBD3	CCTTCTCTTTGCCTTGCTCTT	GCCACTCACAGAACAGCTACC
NF-κB	CTCGCACAAGGAGACATGAA	ACTCAGCCGGAAGGCATTAT
MyD88	TGGTAGTGGTTGTCTCTGATGA	TGGAGAGAGGCTGAGTGCAA
TLR-2	TCACTTGTCTAACTTATCATCCTCTTG	TCAGCGAAGGTGTCATTATTGC
Gapdh	CGGAGTGAACGGATTTGGC	TGCCGTGGGTGGAATCATAC

**Table 4:** The percentages of cecal contents dominant bacteria in Genus level.

Item	Con	BL	CB	CB-BL
*Actinobacillus*	2.78 ± 0.32	1.55 ± 1.12	0.63 ± 0.56	1.39 ± 1.16
*Alloprevotella*	1.04 ± 0.55	3.10 ± 1.95	0.29 ± 0.22	1.41 ± 0.93
*Anaerotruncus*	1.68 ± 0.48	2.03 ± 1.20	2.06 ± 1.75	1.01 ± 0.37
*Anaerovibrio*	0.89 ± 0.57	4.70 ± 2.38	1.30 ± 1.14	0.78 ± 0.40
*Blautia*	1.76 ± 0.33	1.90 ± 0.93	4.27 ± 2.47	1.32 ± 0.29
*Clostridium_sensu_stricto_1*	10.07 ± 2.82	12.79 ± 8.34	17.71 ± 12.80	5.67 ± 0.64
*Coprococcus*	4.94 ± 1.63	3.56 ± 0.89	1.70 ± 0.95	3.37 ± 1.55
*Dorea*	4.23 ± 3.87	1.65 ± 0.83	1.31 ± 2.08	1.47 ± 0.96
*Erysipelotrichaceae_incertae_sedis*	2.20 ± 1.70	4.67 ± 3.98	5.49 ± 2.13	0.68 ± 0.08
*Escherichia-Shigella*	0.18 ± 0.12	4.62 ± 4.45	1.87 ± 1.32	10.75 ± 5.27
*Faecalibacterium*	1.33 ± 1.07 ^b^	3.48 ± 1.68 ^ab^	4.53 ± 3.36 ^ab^	1.20 ± 1.12 ^b^
*Lachnospiraceae_incertae_sedis*	1.54 ± 0.61	2.24 ± 0.56	5.52 ± 3.12	1.87 ± 1.04
*Lachnospiraceae_unclassified*	4.66 ± 1.87	2.28 ± 1.06	1.29 ± 0.55	4.69 ± 0.69
*Lactobacillus*	1.78 ± 0.62 ^b^	3.95 ± 0.81 ^b^	4.32 ± 0.17 ^b^	15.55 ± 2.13 ^a^
*Leeia*	1.61 ± 0.51	2.94 ± 2.27	9.27 ± 6.61	3.49 ± 1.76
*Phascolarctobacterium*	5.18 ± 2.56 ^a^	1.05 ± 0.54 ^b^	0.28 ± 0.23 ^b^	0.60 ± 0.29 ^b^
*Pseudobutyrivibrio*	3.01 ± 1.57	3.22 ± 0.80	4.87 ± 4.70	1.85 ± 0.36
*Roseburia*	1.13 ± 0.68	2.72 ± 0.78	3.33 ± 3.26	1.27 ± 0.53
*Ruminococcaceae_unclassified*	2.44 ± 0.66	4.94 ± 0.50	0.73 ± 0.06	1.23 ± 0.27
*Ruminococcaceae_uncultured*	15.34 ± 3.04	5.03 ± 3.27	4.95 ± 2.61	18.16 ± 3.77
*Streptococcus*	0.72 ± 0.21	3.10 ± 2.76	3.15 ± 0.58	1.91 ± 1.32
*Subdoligranulum*	3.49 ± 1.29	2.45 ± 0.90	2.49 ± 1.08	5.23 ± 2.60
Other	27.94 ± 4.53	21.99 ± 3.56	18.59 ± 7.35	15.04 ± 7.35
Total	100	100	100	100

^a,b,c^ Mean values within a row with different superscript letters are significantly different (*p* < 0.05).

## Data Availability

The data of qRT-PCR, Western blot, SCFAs and microbiota changes used to support the findings of this study are available from the corresponding author upon request.
